# Psychiatric characteristics of older persons with medically unexplained symptoms: A comparison with older patients suffering from medically explained symptoms

**DOI:** 10.1192/j.eurpsy.2020.51

**Published:** 2020-05-20

**Authors:** D. J. C. Hanssen, T. J. W. van Driel, P. H. Hilderink, C. E. M. Benraad, P. Naarding, T. C. Olde Hartman, P. L. B. J. Lucassen, R. C. Oude Voshaar

**Affiliations:** 1 University of Groningen, University Medical Center Groningen, Interdisciplinary Center for Psychopathology of Emotion Regulation (ICPE), Groningen, The Netherlands; 2 SeniorBeter, Practice for Old Age Psychiatry, Gendt, The Netherlands; 3 Radboud University Nijmegen Medical Centre, Department of Geriatric Medicine/Radboudumc Alzheimer Centre, Nijmegen, The Netherlands; 4 Department of Old-age Psychiatry, GGNet, Apeldoorn, The Netherlands; 5 Radboud University Nijmegen Medical Centre, Radboud Institute for Health Sciences, Department of Primary and Community Care, Nijmegen, The Netherlands

**Keywords:** Medically unexplained symptoms, psychiatric characteristics, somatization, somatoform disorder

## Abstract

**Background.:**

Empirical studies on the clinical characteristics of older persons with medically unexplained symptoms are limited to uncontrolled pilot studies. Therefore, we aim to examine the psychiatric characteristics of older patients with medically unexplained symptoms (MUS) compared to older patients with medically explained symptoms (MES), also across healthcare settings.

**Methods.:**

A case–control study including 118 older patients with MUS and 154 older patients with MES. To include patients with various developmental and severity stages, patients with MUS were recruited in the community (*n* = 12), primary care (*n* = 77), and specialized healthcare (*n* = 29). Psychopathology was assessed according to Diagnostic and Statistical Manual of Mental Disorders, Fourth Edition, Text Revision (DSM-IV-TR) criteria (Mini-International Neuropsychiatric Interview) and by dimensional measures (e.g., psychological distress, hypochondriasis, and depressive symptoms).

**Results.:**

A total of 69/118 (58.5%) patients with MUS met the criteria for a somatoform disorder according to DSM-IV-TR criteria, with the highest proportion among patients recruited in specialized healthcare settings (*p* = 0.008). Patients with MUS had a higher level of psychological distress and hypochondriasis compared to patients with MES. Although psychiatric disorders (beyond somatoform disorders) were more frequently found among patients with MUS compared to patients with MES (42.4 vs. 24.8%, *p* = 0.008), this difference disappeared when adjusted for age, sex, and level of education (odds ratio = 1.7 [95% confidence interval: 1.0–3.0], *p* = 0.070).

**Conclusions.:**

Although psychological distress is significantly higher among older patients with MUS compared to those with MES, psychiatric comorbidity rates hardly differ between both patient groups. Therefore, treatment of MUS in later life should primarily focus on reducing psychological distress, irrespective of the healthcare setting patients are treated in.

## Introduction

Medically unexplained symptoms (MUS) are defined as “physical symptoms that have existed for more than several weeks and for which adequate medical examination has not revealed any condition that sufficiently explains the symptoms” [1]. Doctors often feel pressurized to offer unnecessary medical investigations and referrals [2], putting patients at risk for false positive results and iatrogenic damage [3]. In line with this, patients with MUS have approximately twice as much healthcare costs compared to nonsomatizing patients [4], making them a high economic burden to society. Moreover, the presence of physical symptoms in itself is consistently associated with a decreased health-related quality of life, with patients with MUS reporting similar or even lower health-related quality of life rates than patients with medically explained symptoms (MES) [5,6].

A systematic review demonstrated that patients of age 65 and above less frequently report MUS compared to younger age groups, with prevalence rates ranging between 4.6 and 18% [7]. However, lower prevalence rates with increasing age may be an artifact. Physicians might be reluctant to classify symptoms as unexplained out of fear of missing a somatic explanation or MUS are attributed to comorbid somatic diseases, as the prevalence of MES increases with aging [7]. Hence, interpretation of the characteristics of older patients with MUS against a sample of older patients with MES is relevant, especially for clinical care.

As far as we are aware, empirical knowledge on the clinical characteristics of MUS in later life is limited. Two papers derived from an uncontrolled pilot study report that late-life MUS frequently presents itself as a mixture of explained and unexplained physical symptoms [8]; furthermore, in this study, two-third of the older patients with MUS suffered from comorbid psychiatric disorders, most often a major depressive disorder (56%) [9]. This very high comorbidity rate with psychiatric disorders other than a somatoform disorder (SFD) may be explained by the fact that this pilot study has been conducted at an outpatient mental health clinic. This explanation is supported by a prospective study in which the presence of MUS in primary care had a low predictive value for anxiety and depression [10]. However, some community samples also report high comorbidity rates between somatoform, mood, and anxiety disorders [11,12], corresponding to the idea that mental distress is a main driver for help-seeking behavior in case of physical symptoms [13]. Although it is often assumed that severity indicators of MUS, such as the presence of psychiatric comorbidity, differ between healthcare settings, it is unknown whether or not this goes up for older-aged patients.

The primary objective of the present study is to explore the psychiatric characteristics of older patients suffering from MUS with a comparison group of older patients with MES. A secondary objective is to explore whether severity indicators of MUS vary between healthcare settings (community, primary care, or specialized healthcare).

## Methods

### Study design

The Older Persons with Medically Unexplained Symptoms (OPUS) study has been designed as a case–control study including a total of 272 older patients suffering from actual physical symptoms (118 cases suffering from MUS, 154 controls suffering from MES). The main objective of the OPUS study is to examine clinical and care characteristics, as well as consequences of late-life MUS. Baseline data of the OPUS study were collected between September 2011 and March 2014. The local Medical Ethical Committee of the Radboud University Nijmegen Medical Center has approved the OPUS study.

The recruitment process was designed to compose a sample of older patients with MUS in various developmental and severity stages in order to overcome setting-specific findings. Therefore, possible participants with MUS and MES were recruited in the community by advertisements in local newspapers, in primary care, and in secondary healthcare (i.e., specialized mental health clinic for late-life MUS; geriatric department of university hospital). To assist general practitioners (GPs) with selecting possible participants, the top 20% of older frequent attending patients in their own practice were extracted from the GP Information System. Based on this selection, GPs selected those patients meeting our selection criteria (see below) and invited them to participate in the study. This selection method was chosen based on previous research projects on MUS and other high-utilizing patient groups in primary care [14,15]. Primary care patients with MES were also recruited from the frequent attenders list for two reasons. First, we strived for a control group with current physical symptoms with a severity comparable to those of the patients with MUS. Since patients with stable chronic somatic diseases or multimorbidity do not necessarily have current physical symptoms, patients with MES with current physical complaints can more likely be found among frequent attenders. Second, the discrimination between MUS and MES among frequent attenders is most difficult in primary care. Therefore, by selecting the participants using the frequent attenders list, we expected to increase the clinical relevance of the OPUS study.

### Participants

Inclusion criteria for cases were (a) age of 60 years or above and (b) the presence of MUS according to the definition for MUS of the Dutch College of General Practitioners, that is, physical symptoms that have existed for more than several weeks and for which adequate medical examination has not revealed any condition that sufficiently explains the symptoms [1]. We operationalized “several weeks” as at least 3 months. Patients suffering from so-called functional syndromes, that is, fibromyalgia, chronic fatigue syndrome, irritable bowel syndrome, or a whiplash syndrome, were also included as patients with MUS [16]. As part of the study protocol, the unexplained nature of the MUS-patient’s symptoms was checked by either a comprehensive assessment conducted by a geriatrician (*n* = 70) or an additional chart review of the GP for patients who refused this geriatric assessment (*n* = 48) but agreed with the other study procedures.

Exclusion criteria for both patient groups were (a) presence of a primary psychotic disorder, (b) established or suspected diagnosis of dementia, (c) suffering from terminal illness, (d) insufficient mastery of the Dutch language, and (e) auditory or visual impairment interfering with reliable data collection.

**Figure 1.**
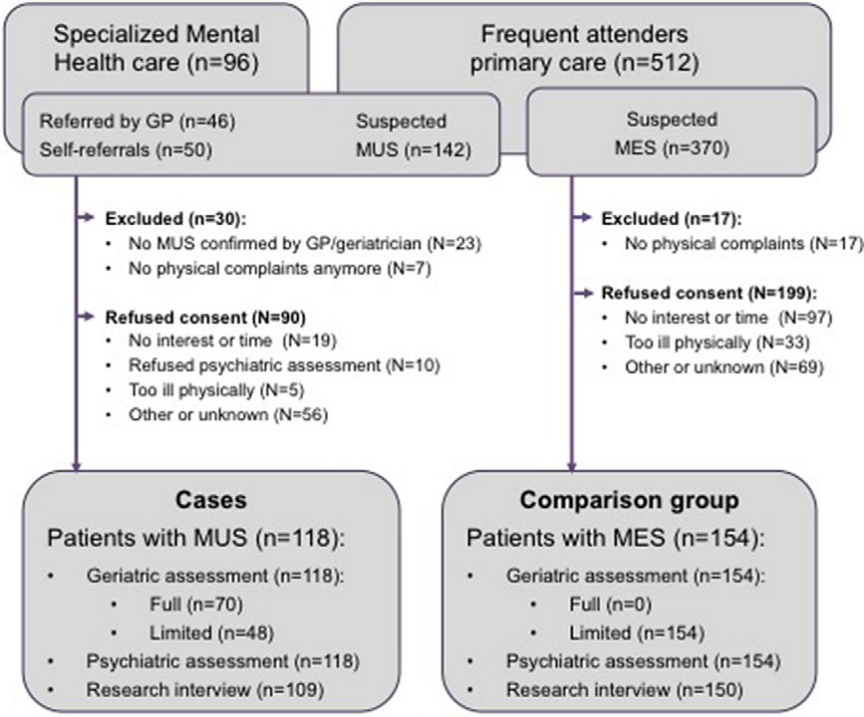
Recruitment of participants.

All participants of the OPUS study gave written informed consent. Figure 1 presents the results of the recruitment process, which has been described in more detail elsewhere [17]. Of the 118 patients with MUS, 12 (10.2%) were recruited in the community, 77 (65.3%) in primary care, and 29 (24.6%) in specialized healthcare.

### Procedures of OPUS study

All participating patients with MUS were offered a multidisciplinary diagnostic procedure, consisting of a comprehensive physical assessment by a geriatrician (C.E.M.B.) and semi-structured interviews by an old-age psychiatrist (P.H.H.) and psychologist (T.J.W.v.D.). Subsequently, the participant filled out a number of questionnaires. After the diagnostic procedure, a researcher (D.J.C.H.) visited the patient at home to examine social and cognitive functioning in more depth.

If patients with MUS refused to participate in the multidisciplinary diagnostic procedure, but nevertheless agreed to participate in the OPUS study, the researcher (D.J.C.H., supervised by P.N.) performed two home-visits (40.7%; 48/118). During the additional home visit, the researcher administered all instruments used by the multidisciplinary team (see below), with the exception of the geriatric measurement Cumulative Illness Rating Scale-Geriatric, for which training was deemed insufficient to ensure reliable data collection. Patients with MES always received two home-visits in which all research instruments were administered, similarly to patients with MUS who refused the diagnostic workup at the specialized mental health clinic.

### Measurements

For the present study on psychiatric comorbidity, we explored sociodemographic characteristics, psychopathology, severity of the primary physical complaint, and severity indicators of MUS.

*Demographic characteristics* were determined by self-report questions based on the Older Persons and Informal Caregivers Survey [18]. The highest level of education was categorized in low, middle, or high.

We recorded the *primary physical complaint* of the patient and assessed duration of the complaint (in years). The severity of the primary physical complaint was assessed with 10-cm visual analogue scales (VASs) (average severity over the past month as well as highest severity in the past month).

The Mini-International Neuropsychiatric Interview (MINI) [19] was applied to assess *psychopathology conform DSM-IV criteria.* Specifically, questions on depressive disorders, anxiety disorders, obsessive compulsive disorder, alcohol and drugs dependence or abuse, and SFDs were assessed with this semi-structured diagnostic interview.

The Brief Symptom Inventory-53 (BSI-53) was administered to assess *psychopathology from a dimensional perspective.* The BSI-53 is an abbreviated version of the Symptom Checklist 90-item version (SCL-90) [20]. The BSI-53 has 53 items to be rated on a 5-point scale (range 0–4) and assesses nine domains, that is, somatization, obsession–compulsion, interpersonal sensitivity, depression, anxiety, hostility, phobic anxiety, paranoid ideation, and psychoticism (i.e., positive symptoms of psychosis, and social withdrawal) [21], without loss of information compared to the SCL-90 [22] and with good internal consistency and test–retest reliability [23]. The *somatization* subscale consists of seven items referring to the severity of physical symptoms, that is, dizziness, chest pain/discomfort, nausea, shortness of breath, hot flushes, paresthesia, and faintness/general weakness.

*Severity of depressive symptoms* was measured by the 30-item self-rating Inventory of Depressive Symptomatology, which has adequate psychometric properties [24]. The sum score ranges from 0 to 84.

*Severity of anxiety symptoms* was assessed with the anxiety section of the Hospital Anxiety and Depression Scale (HADS) [25]. This anxiety subscale comprises seven 4-point Likert-scaled items and mainly covers symptoms of generalized anxiety and panic attacks. The basic psychometric properties of the HADS were considered as quite good to very good [26].

The Whitely Index (WI) [27] was used to measure *hypochondriasis* based on 14 statements that have to be rated as yes or no.

### Severity indicators of MUS

As potential severity indicators, we selected (a) severity of the primary complaint (VAS), (b) duration of the primary complaint in years, (c) severity of hypochondriasis (WI), (d) level of somatization (subscale of the BSI-53), (e) presence of an SFD according to DSM-IV-TR criteria (MINI), and (f) presence of a psychiatric disorder other than an SFD (MINI).

### Statistical analyses

Sociodemographic and psychiatric characteristics of patients with MUS were compared with patients with MES using chi-square tests (categorical variable) or Student’s *t* tests (continuous variables). Subsequently, psychiatric characteristics were compared between patients with MUS and patients with MES. Logistic regression analyses were applied to examine whether patients with MUS had higher odds on the presence of any or a specific psychiatric disorder (dependent variable), adjusted for age, sex, and level of education. Dimensional measures were compared between both groups by Student’s *t* test as well as by analysis of covariance, adjusted for age, sex, and level of education. Due to the expected high interference between comorbid psychiatric disorders and psychological distress, we also performed a sensitivity analysis in which we compared all dimensional measures between patients with MUS and MES who did not have a psychiatric disorder (other than an SFD).

Severity indicators of MUS were compared between patients recruited in the community, in primary care, or in specialized medical care with chi-square tests (categorical variable) or analysis of variance (continuous variables).

Despite the high number of comparisons, *p* values equal to or less than 0.05 are considered significant to prevent type I errors, that is, rejecting potentially relevant differences for future studies (because of the lack of controlled studies in this area). However, as correcting for multiple comparisons may increase the risk of type II error, we present all individual *p* values [28].

## Results

### General characteristics

The descriptive characteristics of patients with MUS and MES are presented in Table 1. The severity of the primary complaint did not differ between the two groups, although patients with MUS suffered significantly longer from their physical symptoms. Although the primary physical complaint significantly differed between both groups (Table 1), pain was most frequently reported in both groups.

**Table 1.** Demographic and basic clinical characteristics of patients with MUS and MES.

		MUS	MES	
		(*n* = 118)	(*n* = 154)	Statistics
Demographics				
Age (years)	Mean (SD)	70.5 (6.7)	73.4 (7.7)	*t* = −3.2, df = 270, *p* < 0.001
Female sex	*n* (%)	76 (64.4)	67 (43.5)	*χ* ^2^ = 11.7, df = 1, *p* = 0.001
Level of education				*χ* ^2^ = 3.2, df = 2, *p* = 0.205
Low	*n* (%)	29 (26.9)	27 (17.8)	
Middle	*n* (%)	49 (45.4)	80 (52.6)	
High	*n* (%)	30 (27.8)	45 (29.6)	
Stable partnership	*n* (%)	66 (60.6)	92 (60.5)	*χ* ^2^ < 0.1, df = 1, *p* = 0.997
Primary physical complaint				
Pain	*n* (%)	71 (60.2)	69 (44.8)	*χ* ^2^ = 6.3, df = 1, *p* = 0.012^a^
Dizziness	*n* (%)	1 (0.8)	4 (2.7)	
Palpitations	*n* (%)	4 (3.8)	3 (1.9)	
Shortness of breath	*n* (%)	1 (0.8)	10 (6.8)	
Constipation	*n* (%)	2 (1.9)	2 (1.4)	
Nausea	*n* (%)	1 (0.8)	1 (0.7)	
Fatigue	*n* (%)	7 (6.7)	7 (4.7)	
Problems with sleeping	*n* (%)	1 (0.8)	2 (1.4)	
Diffuse, fluctuating symptoms	*n* (%)	3 (2.9)	31 (20.9)	
Other	*n* (%)	13 (12.5)	19 (12.8)	
Severity primary complaint (VAS)				
Average past month	Mean (SD)	4.9 (1.8)	4.6 (2.6)	*t* = 0.9, df = 238, *p* = 0.393
Most severe past month	Mean (SD)	6.3 (2.1)	6.0 (3.2)	*t* = 0.7, df = 234, *p* = 0.504
Duration of primary complaint (years)	Median (IQR)	5.0 (9.5)	2.0 (9.8)	*t* = 2.8, df = 210, *p* = 0.006^b^

Abbreviations: IQR, interquartile range; MES, medically explained symptoms; MUS, medically unexplained symptoms; SD, standard deviation; VAS, visual analogue scale.

a Chi-square testing pain (yes/no); other symptoms lumped together.

b The *t* test conducted after Ln transformation to achieve a normal distribution.

### Psychiatric characteristics

Of the 118 patients with MUS, 69 (58.5%) met the criteria for an SFD according to DSM-IV-TR criteria. The individual SFDs identified were pain disorder (*n* = 26), undifferentiated SFD (*n* = 36), hypochondriasis (*n* = 7), and SFD Not Otherwise Specified (*n* = 3). Three patients had two different SFDs (included in the individual numbers described above), and none of the participants had a somatization disorder. None of the patients with MES met the criteria for an SFD.

Patients with MUS had significantly more often a psychiatric disorder other than SFD compared to patients with MES (Table 2). Of the specific psychiatric disorders, anxiety and adjustment disorders were significantly more frequent among patients with MUS compared to patients with MES, whereas depressive disorder was not. None of these differences, however, remained statistically significant when adjusted for age, sex, and level of education.

**Table 2.** Categorical and dimensional measures of psychopathology in patients with MUS compared to patients with MES.

		Patients with MUS	Patients with MES	Statistics	
		(*n* = 118)	(*n* = 154)	Univariate	Multivariate^a^
Psychopathology (MINI)					
Somatoform disorder	*n* (%)	69 (58.5)	–	*p* < 0.001	
Other psychiatric disorders	*n* (%)	50 (42.4)	38 (24.8)	OR = 2.2 [1.3–3.7], *p* = 0.002	OR = 1.7 [1.0–3.0], *p* = 0.070
Mood disorder	*n* (%)	31 (26.3)	32 (20.9)	OR = 1.3 [0.8–2.4], *p* = 0.302	OR = 1.0 [0.5–1.9], *p* = 0.986
Anxiety disorder	*n* (%)	22 (18.6)	13 (8.5)	OR = 2.5 [1.2–5.1], *p* = 0.016	OR = 1.8 [0.8–4.0], *p* = 0.131
Substance use disorder	*n* (%)	6 (5.1)	3 (2.0)	OR = 2.7 [0.6–10.9], *p* = 0.170	OR = 2.1 [0.5–9.3], *p* = 0.347
Adjustment disorder	*n* (%)	4 (3.4)	–	*p* = 0.015^b^	n.a.
Psychopathology dimensions					
Hypochondriasis (Whitely Index)	Mean (SD)	4.3 (2.9)	2.2 (2.4)	*t* = 6.3, df = 260, *p* < 0.001	*F* = 32.0, df = 1,253, *p* < 0.001
Depressive symptoms (IDS)	Mean (SD)	20.8 (12.0)	15.2 (9.2)	*t* = 4.2, df = 247, *p* < 0.001	*F* = 8.2, df = 1,224, *p* = 0.005
General anxiety (HADS-A)	Mean (SD)	5.3 (4.0)	3.4 (3.6)	*t* = 3.7, df = 236, *p* < 0.001	*F* = 12.0, df = 1,235, *p* = 0.001
BSI-53 total score	Mean (SD)	0.57 (0.50)	0.42 (0.38)	*t* = 2,6, df = 244, *p* = 0.011	*F* = 5.1, df = 1,232, *p* = 0.025
Somatization	Mean (SD)	0.80 (0.65)	0.52 (0.50)	*t* = 3.9, df = 244, *p* < 0.001	*F* = 18.1, df = 1,232, *p* < 0.001
Obsession–compulsion	Mean (SD)	0.70 (0.72)	0.65 (0.57)	*t* = 0.6, df = 244, *p* = 0.513	*F* = 0.6, df = 1,232, *p* = 0.445
Interpersonal sensitivity	Mean (SD)	0.49 (0.58)	0.40 (0.52)	*t* = 1.3, df = 244, *p* = 0.173	*F* = 0.4, df = 1,232, *p* = 0.527
Depression	Mean (SD)	0.59 (0.70)	0.40 (0.54)	*t* = 2.4, df = 244, *p* = 0.016	*F* = 3.2, df = 1,232, *p* = 0.077
Anxiety	Mean (SD)	0.58 (0.72)	0.32 (0.49)	*t* = 3.3, df = 244, *p* = 0.001	*F* = 6.8, df = 1,233, *p* = 0.010
Hostility	Mean (SD)	0.34 (0.39)	0.29 (0.32)	*t* = 1.2, df = 245, *p* = 0.232	*F* = 1.6, df = 1,233, *p* = 0.207
Phobic anxiety	Mean (SD)	0.43 (0.59)	0.29 (0.45)	*t* = 2.1, df = 245, *p* = 0.036	*F* = 2.7, df = 1,233, *p* = 0.104
Paranoid ideation	Mean (SD)	0.52 (0.58)	0.40 (0.53)	*t* = 1.6, df = 245, *p* = 0.113	*F* = 1.1, df = 1,233, *p* = 0.286
Psychoticism	Mean (SD)	0.49 (0.59)	0.35 (0.45)	*t* = 2.2, df = 245, *p* = 0.032	*F* = 4.6, df = 1,233, *p* = 0.034

Abbreviations: BSI-53, Brief Symptom Inventory-53 item version; HADS-A, Hospital Anxiety Depression Scale-Anxiety subscale; IDS, Inventory of Depressive Symptoms; MES, medically explained symptoms; MINI, Mini-International Neuropsychiatric Interview; MUS, medically unexplained symptoms; SD, standard deviation.

a Adjusted for age, sex, and level of education (low, middle, or high).

b Logistic regression not applicable, and *p* value based on Fisher’s exact test.

Considering the dimensional measures of psychopathology (Table 2), patients with MUS had a significantly higher severity of overall psychopathology (BSI-53 sum score) compared to patients with MES. Of the subscales of the BSI-53, however, only somatization, anxiety, and psychoticism were statistically different between the two groups with higher scores for the patients with MUS. With respect to the disorder-specific severity scales, patients with MUS had a significantly higher level of depression, anxiety, and hypochondriasis compared to patients with MES, when adjusted for age, sex, and level of education.

The sensitivity analyses were conducted on 68 patients with MUS and 115 patients with MES who had no psychiatric disorder (other than an SFD). These analyses revealed that patients with MUS still had a higher level of psychopathology compared to patients with MES with respect to somatization (BSI-53 subscale: *F* = 9.8, df = 1,158, *p* = 0.002), hypochondriasis (WI: *F* = 37.2, df = 1,169, *p* < 0.001), and depression (Inventory of Depressive Symptoms: *F* = 5.5, df = 1,160, *p* = 0.020).

### Severity of MUS

The severity of hypochondriasis and presence of an SFD differed across the recruitment settings (*F* = 6.4, df = 2,106, *p* = 0.002 and *χ* ^2^ = 9.7, df = 2, *p* = 0.008, respectively, see Table 3). Post hoc tests did not reveal any significant differences between patients recruited in the community and those recruited in primary care. Nonetheless, least significant difference post hoc test showed that patients recruited in specialized healthcare scored significantly higher on hypochondriasis compared to those recruited in primary care (*p* = 0.001) and in the community (*p* = 0.035). The proportion of patients suffering from an SFD differed only between those recruited in specialized healthcare and primary care (*p* = 0.002).

**Table 3.** Severity indicators of somatization in patients with MUS (*n* = 118) stratified by recruitment setting.

		Total sample	Community	Primary care	Specialized healthcare	
		(*n* = 118)	(*n* = 12)	(*n* = 77)	(*n* = 29)	Statistics
Severity primary complaint (VAS)						
Average past month	Mean (SD)	4.9 (1.8)	4.2 (1.6)	5.0 (1.9)	4.7 (1.5)	*F* = 1.13, df = 2,98, *p* = 0.324
Most severe past month	Mean (SD)	6.3 (2.1)	5.2 (2.4)	6.6 (2.0)	6.0 (1.9)	*F* = 2.36, df = 2,96, *p* = 0.099
Duration of complaint (years)	Median (IQR)	5.0 (9.5)	5.0 (7.5)	5.0 (13.0)	5.0 (11.0)	*F* = 1.41, df = 2,93, *p* = 0.467^a^
Hypochondriasis (Whitely Index)	Mean (SD)	4.3 (2.9)	4.0 (3.2)	3.8 (2.7)	6.1 (3.0)	*F* = 6.35, df = 2,106, *p* = 0.002
Somatization scale (BSI-53)	Mean (SD)	0.81 (0.65)	1.05 (1.00)	0.74 (0.56)	0.96 (0.71)	*F* = 1.44, df = 2,89, *p* = 0.242
Presence of a somatoform disorder	*n* (%)	69 (58.5)	7 (58.3)	38 (49.4)	24 (82.8)	*χ* ^2^ = 9.7, df = 2, *p* = 0.008
Presence of a psychiatric disorder	*n* (%)	39 (33.1)	3 (25.0)	32 (41.6)	15 (51.7)	*χ* ^2^ = 2.5, df = 2, *p* = 0.280

Abbreviations: BSI-53, Brief Symptom Inventory-53 item version; IQR, interquartile range; MUS, medically unexplained symptoms; SD, standard deviation; VAS, visual analogue scale.

a Based on Ln values as the variable “duration of primary complaint” had a skewed distribution.

## Discussion

### Main findings

Older patients with MUS had, as expected, higher levels of depressive symptoms, anxiety symptoms, hypochondriasis, and psychoticism compared to older patients with MES, but this was not reflected by a significantly higher prevalence of psychiatric disorders according to DSM-IV-TR criteria (except the presence of SFDs). Still, at least two out of five older patients with MUS suffered from comorbid psychiatric disorders that require treatment. As expected, this is especially relevant for older patients with MUS within specialized healthcare settings, since these patients have more severe hypochondria and more often have an SFD compared to primary care patients and patients from the community.

### Psychiatric characteristics

Two-thirds of older patients with MUS met the criteria for an SFD according to DSM-IV-TR criteria. Moreover, patients with MUS had significantly more often a psychiatric disorder other than an SFD compared to patients with MES. Although all individual psychiatric disorders were more frequently identified among MUS compared to patients with MES, only the proportion of anxiety disorders and adjustment disorders achieved statistical significance. This suggests that in later life comorbid anxiety disorders are more specific for MUS than a depressive disorder.

In our sample of older patients with MUS, only one out of four patients had a comorbid depressive disorder. This contrasts with a pilot study of older patients with MUS referred to specialized mental healthcare, among which more than half of the patients had a comorbid depressive disorder [9].

The prevalence rate of depressive disorder did not differ between patients with MUS and MES in our study, as one out of five patients with MES also suffered from a major depressive disorder. Our recruitment process, that is, the frequent attenders method, can explain this relatively high proportion of patients with a depressive disorder among patients with MES. It is known that the prevalence of psychiatric disorders is increased among primary care patients who frequently visit their GP as well as in patients with specific chronic somatic diseases such as chronic obstructive pulmonary disease, inflammatory bowel disease, or diabetes [29–31]. Among older patients with MES, GPs might not recognize MUS resulting in a false classification of depression. This fits with the fact that depression in later life often has a more somatic presentation [32] and the fact that late-life depression amplifies the subjective severity of somatic symptoms. From a clinical perspective, this finding is important as comorbid anxiety or depressive disorders are associated with higher functional impairment levels [11].

The higher proportion of comorbid psychiatric disorders in patients with MUS compared to MES is also reflected by a higher BSI sum score reflecting overall psychopathology, as well as by higher scores on the subscales depression, anxiety, phobia, and somatization. This is in line with comorbid psychopathology levels reported in younger patients with MUS, as up to 60% of patients have clinically relevant levels of comorbid symptoms of anxiety and/or depression [4]. Although the BSI-53 measures past-week severity of psychopathology, the specific subscales with elevated scores may point to an important role for the personality trait neuroticism. Neuroticism has been related to somatization in younger patients [14]. In a previous study, we have also found that older patients with MUS had a higher level of neuroticism compared to primary care control group, although not to patients with MES [33].

### Severity indicators of MUS

Our recruitment procedures ensure inclusion of the whole spectrum of patients with MUS, as patients were recruited by self-referral, by screening frequent attenders in primary care, and by those referred to secondary mental healthcare due to a severe level of functional limitations. Nonetheless, of the a priori selected severity indicators, only the severity of hypochondriasis and the proportion of patients meeting the criteria for an SFD were significantly higher in specialized healthcare settings. In fact, these findings are in line with DSM-5 in which the distinction between explained and unexplained symptoms has been abandoned and health anxiety (hypochondriasis) is considered a much more relevant construct [34]. Due to small patient numbers recruited in the community, however, the lack of any difference between patients recruited in the community and those recruited in primary care should be interpreted cautiously.

### Methodological considerations

The OPUS study has several strengths. First, by including 118 older patients with MUS, the OPUS study has built the largest cohort of older patients with MUS hitherto. Second, experienced healthcare professionals instead of research assistants extensively assessed patients at baseline. Third, psychopathology was assessed categorically by formal classification according to DSM-IV-TR criteria using semi-structured interviews, as well as dimensionally by administering self-report symptom questionnaires. Fourth, even though some of the group numbers are small, we still have included patients across three healthcare echelons thereby covering the whole severity spectrum of MUS. Finally, the comparison group of patients with MES had a comparable severity level of the primary somatic complaints, indicating good matching of both groups. Therefore, differences between the two groups of patients with MUS and MES are highly relevant for clinical practice, as people only present themselves to clinicians when having symptoms. Comparisons with community-dwelling healthy seniors or older patients with multimorbidity without actual physical complaints would be interesting from a theoretical perspective, but are less relevant for clinicians.

However, limitations should also be addressed. First, a case–control design was selected over a cohort study because of its suitability for exploratory research questions and the relatively small sample size needed, as a full diagnostic workup is labor-intensive. Nonetheless, a population-based cohort study would be preferred because of its ability to identify (psychiatric) determinants prior to the onset of MUS (i.e., predictors of MUS). Second, it might be possible that physical symptoms in the MES group have been wrongly attributed to somatic comorbidity and/or signs of old age, leading to possible underreporting of SFDs in our research population. Nonetheless, we consider this unlikely based on our clear definitions for MUS and MES, the extensive somatic screening for each of the participants, and previous findings in our pilot study showing good inter-rater reliability between geriatricians in classifying somatic symptoms as completely explained, partially explained, or unexplained [8]. Last, our study was set up before DSM-5 criteria for somatic symptom disorders were launched. Although the DSM-5 does not address clinical issues related to MUS (by leaving the distinction between explained and unexplained symptoms), the lack of the DSM-5 classification is a clear limitation. As somatic symptom disorders are based on excessive behavior, emotions, or cognitions related to physical symptoms, we might try to approach these criteria with the OPUS data. However, we feel that our dataset is too limited to do so as we only have quantified hypochondriasis (health anxiety) and illness cognitions [35], but not excessive behavior.

### Clinical implications

Our current results suggest that older patients with MUS, regardless of healthcare setting, might benefit from treatment of psychological distress, even when psychiatric comorbidity is absent. However, treatment of these symptoms within primary care or referral to a psychologist or psychiatrist by GPs is rather exceptional [36], especially for older patients. Qualitative studies have demonstrated that patients as well as GPs interpret low mood and worry in patients with MUS as an individual response to their circumstances instead of being a circumscribed problem in need of care [37]. Treatment of relatively mild complaints could, in our opinion, be provided in primary care, for example, by trained mental health nurses (also to avoid potential financial barriers), whereas treatment of severe complaints and/or psychiatric comorbidity could take place in mental health institutions. To achieve optimal treatment, we believe that psychological treatment should be carried out in close collaboration with the older patient’s GP and/or geriatrician.

## Data Availability

The data that support the findings of this study are available from the corresponding author upon reasonable request.
